# Effect of interfacial surface treatment on bond strength of particulate-filled composite to short fiber-reinforced composite

**DOI:** 10.1080/26415275.2022.2070489

**Published:** 2022-05-05

**Authors:** L. Lassila, J. Tuokko, A. Suni, S. Garoushi, P. K. Vallittu

**Affiliations:** a Department of Biomaterials Science and Turku Clinical Biomaterials Center – TCBC, Institute of Dentistry, University of Turku, Turku, Finland; b City of Turku Welfare Division, Oral Health Care, Turku, Finland

**Keywords:** Short-fiber reinforced composite, surface treatment, flowable composite, shear bond strength

## Abstract

**Objective:**

The aim was to investigate the effect of different interfacial surface treatments on the shear bond strength (SBS) between a short fiber-reinforced flowable composite (SFRC) and a particulate-filled flowable composite (PFC). In addition, SBS between two successive layers of similar materials was evaluated.

**Materials and methods:**

One-hundred and forty-four specimens were prepared having either SFRC (everX Flow) as a substructure composite and PFC (G-aenial Flo X) as a surface composite or having one of the two materials as both substructure and surface layer. Eight groups of specimens were created (*n* = 18/per group) according to the interfacial surface protocol used. Group 1: no treatment; Group 2: ethanol one wipe; Group 3: ethanol three wipes; Group 4: phosphoric acid etching + bonding agent; Group 5: hydrofluoric acid etching + bonding agent; and Group 6: grinding + phosphoric acid etching. Group 7: only PFC layers and Group 8 (control) only SFRC layers without any surface treatment. After one-day storage (37 °C), SBS between surface and substructure composite layers was measured in a universal testing machine, and failure modes were visually analyzed. SEM was used to examine the bonding surface of the SFRC composite after surface treatment. SBS values were statistically analyzed with a one-way analysis of variance (ANOVA) followed by the Tukey HSD test (α = .05).

**Results:**

The SBS between successive SFRC layers (Group 8) was statistically (*p* < .05) the highest (43.7 MPa) among tested groups. Surface roughening by grinding followed by phosphoric acid etching (Group 6) resulted in a higher SBS (28.8 MPa) than the remaining surface treatments.

**Conclusion:**

Flowable composite with glass fibers (everX Flow) showed higher interlayer SBS compared to PFC flowable composite. Interfacial surface roughness increases the bonding of PFC to the substructure of SFRC.

## Introduction

Direct composite restoration, also known as particulate-filled composite (PFC) restoration, is a common restorative procedure for treating lost tooth structure. It has been reported that general dental practitioners in public dental facilities spend more than half their time applying direct composite restorations [1]. Aside from the capability to adhere to tooth structures *via* bonding systems, direct PFC composite restorations are less expensive than indirect ceramic/composite restorations [2]. The application of direct PFC composites has expanded to include not just posterior intra-coronal restorations, but also extra-coronal restorations [2]. Nevertheless, mechanical properties and polymerization shrinkage are still issues with contemporary PFCs. In small and medium-sized cavities, PFC restorations have shown satisfactory overall clinical performance, with annual failure rates ranging from 1 to 3 percent [3,4]. However, the clinical performance of PFC restorations is clearly associated with restoration size. Large PFC restorations have proven to be more likely to fail due to fractures, resulting in shorter lifespans [3,4].

The reinforcing phase of PFCs has been thoroughly studied with the purpose of improving their viability for application in high-stress areas. Efforts have been made to alter the type of filler used, as well as the size and silanization of the filler [5–7]. Among the strategies investigated, reinforcing the PFC with short glass fibers has proven to be one of the most successful [6,8,9]. Short fibers improved the material's facility to withstand crack propagation and reduced the stress intensity at the crack tip, where a crack spreads in an unstable way [10]. As a result, an enhancement in composite toughness was observed [10,11]. In 2019, the flowable version of short fiber-reinforced composite (SFRC) was introduced with the promise of easy handling and better adaptability in limited spaces [12,13]. Compared to PFC, this SFRC was found to have enhanced mechanical properties in terms of fracture toughness and fatigue resistance [12–14]. It should be taken into account that SFRC is recommended to be used as bulk base or core foundation and should not be used as a top surface layer. According to the manufacturers’ recommendations, SFRC should be covered with a layer (1–2 mm) of flowable or packable PFC to ensure sufficient esthetic appearance.

Many *in vitro* studies have looked at bi-layered composite structures using SFRC as the substructure and PFC as the top surface layer [14–17]. In these investigations, SFRC was used to reinforce extensive direct composite restorations as substructure foundations by supporting the PFC layer and acting as a crack prevention layer. However, there is little knowledge regarding the interlayer bond strength between SFRC and PFC. A previous investigation showed that ethanol application might cause some dissolution of the polymer matrix of fiber-reinforced composite, resulting in increasing surface roughness [18]. The question arises as to whether one may use ethanol wiping to expose the fibers from the surface of SFRC. This might improve the interlayer bonding by means of micromechanical interlocking.

Accordingly, this research aimed 1. to investigate the effect of different interfacial surface treatments on the shear bond strength (SBS) between SFRC and PFC and 2. to determine the SBS of two successive layers of similar materials.

## Materials and methods

Two commercially available flowable composites, one PFC (G-aenial Flo X) and one SFRC (everX Flow) were used in this study (Table 1).

**Table 1.** The flowable resin composites used in the study.

Material (shade/code)	Manufacturer	Composition
G-aenial Flo X (A3/PFC)	GC Corp, Tokyo, Japan	UDMA, dimethacrylate co-monomers. 69 wt% Barium glass fillers in nanometer scale (av. Ø 700 nm)
everX Flow (Bulk shade/SFRC)	GC Corp, Tokyo, Japan	Bis-EMA, TEGDMA, UDMA. 70 wt% Short glass fiber (Ø 6 µm & barium glass fillers Ø 700 nm)

Bis-EMA, Ethoxylated bisphenol-A-dimethacrylate; TEGDMA, triethylene glycol dimethacrylate; UDMA, urethane dimethacrylate; wt%, weight percentage.

### Specimen preparation

A total of 48 acrylic blocks were prepared in cold cure auto-polymerized acrylic resin (Vertex-Dental B.V., Zeist, The Netherlands). Three standardized holes (diameter = 6 mm, depth = 4 mm) were prepared in each block using a bench drill press machine (DP2000A, Rexon Industrial Corporation, Ltd., Taichung, Taiwan). The holes, later to be filled with the substructure composite, were drilled so that they were in an equal distance in relation to each other. A total of 144 specimens were then fabricated having either SFRC as a substructure composite and PFC as a surface composite or having the same material as both substructure and surface composite. Specimens were divided into 8 groups (*n* = 18/per group) according to the used treatment protocol for substructure composite surface (Table 2).

**Table 2.** Test groups and their interfacial surface treatments.

Group	Substructure/surface layer	Interfacial surface treatment
1	SFRC/PFC	Immediate application without treatment
2	SFRC/PFC	Ethanol one wipe for 10 s
3	SFRC/PFC	Ethanol three wipes for 30 s
4	SFRC/PFC	Phosphoric acid etching + bonding agent
5	SFRC/PFC	Hydrofluoric acid etching + bonding agent
6	SFRC/PFC	Grinding (320 grit) + phosphoric acid etching
7	PFC/PFC	Immediate application without treatment (PFC)
8	SFRC/SFRC	Immediate application without treatment (SFRC, control)

SFRC composite was used as a substructure in Groups 1–6. SFRC was applied into the drilled holes in a bulk increment of 4 mm, flattened (plastic instrument) and light cured (Elipar TM S10, 3 M ESPE, Germany) for 40 s from the top surface. The wavelength of the light was between 430 and 480 nm and light intensity was 1200 mW/cm^2^ (Marc Resin Calibrator, BlueLight Analytics Inc., Canada). After curing, the surface of SFRC was manipulated with different surface treatment protocols before the application of surface PFC (Table 2). In Group 1, no surface treatment was applied. In Group 2, the substructure composite surface was exposed to ethanol (concentration 99%) for 10 s (one wipe). In Group 3, the composite surface was exposed to ethanol for 30 s followed by air-drying for 10 s (three wipes). In Group 4, the composite surface was etched with 37% phosphoric acid (Scotchbond Universal Etchant, 3 M ESPE, USA) for 10 s, then rinsed with water for 10 s and air dried for 5 s. Etching was followed by the application of bonding agent (G-Premio Bond, GC Corp, Tokyo, Japan). The bonding agent was abundantly placed on the surface for 40 s. Then the excess was removed by blowing with air for 5 s followed by light curing (Elipar TM S10) for 10 s.

In Group 5, the composite surface was acid-etched by 4.5% hydrofluoric acid (Ivoclar Vivadent, Schaan, Liechtenstein) for 60 s followed by rinsing with water and air-drying. Subsequently, the composite surface was treated with the bonding agent as in Group 4. In Group 6, the composite surface was ground on 320 grit silicon carbide paper using an automatic grinding machine (Rotopol-1; Struers A/S, Copenhagen, Denmark) and then acid-etched as in Group 4. In groups 7 and 8 (control), the cured substructure composite (SFRC or PFC) was immediately covered with a surface layer of the same material and without any surface treatment.

**Figure 1.**
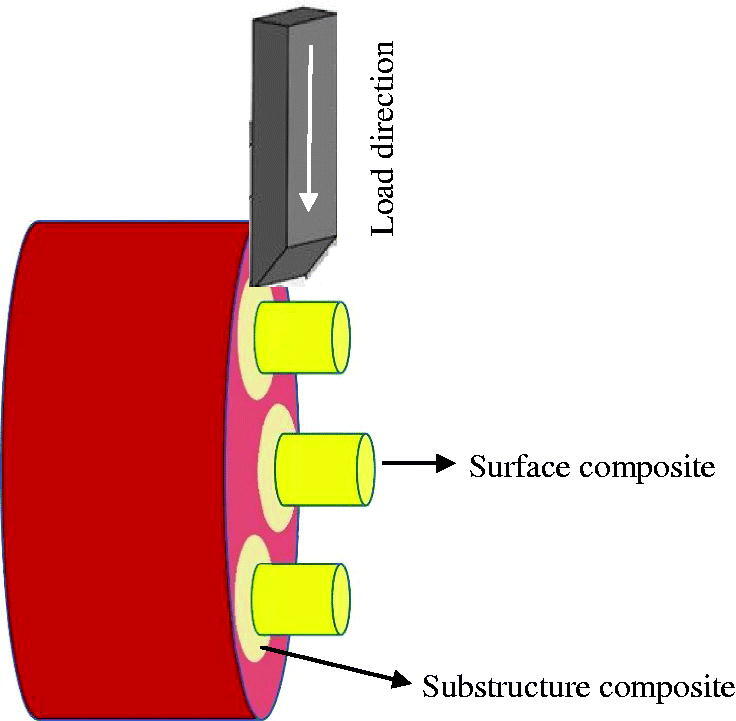
Schematic figure of the debonding test (shear bond strength test) setup.

To allow application of the surface PFC layer (the stub), a transparent polyethylene mold (inner diameter 3.6 mm and height 3 mm) was positioned centrally on the flat substructure SFRC surface. The PFC was applied (2 mm thick layer) and light-cured through the mold from the top and lateral curved surfaces for 40 s (Elipar TM S10). Then, the mold was carefully removed, and specimens (Figure 1) were stored for 24 h in water (37 °C) before testing.

### Interlayer debonding test

The strength of the bond between the surface and substructure composite layers was measured using a shear bond strength test (Figure 1). The specimens were fixed in a mounting jig (Bencor Multi-T shear assembly, Danville Engineering Inc., San Ramon, CA, USA) and a shearing rod was placed parallel to and against the interface between the two composite layers. Then, at room temperature (23 ± 1 °C) and a crosshead speed 1.0 mm/min, a universal testing machine (Model LRX, Lloyd Instruments Ltd., Fareham, England) was utilized to load the specimens until failure. Data were recorded by PC software (Nexygen, Lloyd Instruments Ltd., Fareham, England). The bond strength was calculated by dividing the maximum load at failure (N) with the bonding area (mm^2^). The results were recorded in megapascal (MPa).

### Microscopic analysis

Failure modes of specimens were visually examined and analyzed using a stereomicroscope at magnification force of 15 (Wild M3Z, Wild Heerbrugg, Switzerland). The failure modes were then classified either as adhesive failures between the two composite layers or as cohesive failures within either the substructure or the surface composite.

The effect of surface treatment on SFRC was evaluated using scanning electron microscopy (SEM) (JSM 5500, Jeol, Japan). Before examination, specimens were coated with a gold layer in a vacuum evaporator using a sputter coater (BAL-TEC SCD 050 Sputter Coater, Balzers, Liechtenstein).

### Statistical analysis

Data were statistically analyzed using one-way analysis of variance (ANOVA) followed by the Tukey HSD test (α = .05) to test for differences in shear bond strength between the groups using SPSS version 23 (SPSS, IBM Corp., NY, USA).

## Results

**Figure 2.**
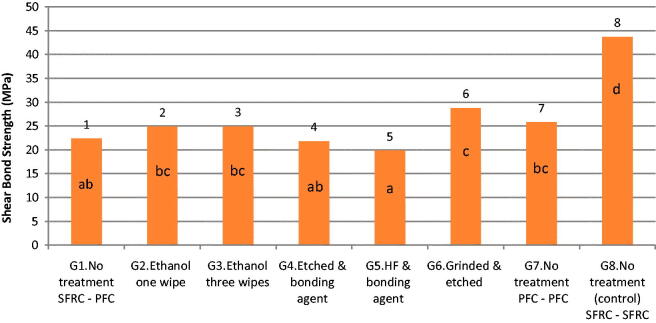
Shear bond strength (mean values and standard deviations; MPa) of the tested groups (*n* = 18). Same letters indicate no statistically significant differences between groups.

The interlayer shear bond strength results are presented in Figure 2. One-way ANOVA demonstrated a significant difference between the groups (*p* < .05). Only grinding followed by phosphoric acid etching (Group 6) resulted in statistically higher shear bond strength (28.8 MPa) than Group 1 (without surface treatment) (22.3 MPa). Etching with hydrofluoric acid followed by application of bonding agent (Group 5) resulted in lowest the interlayer shear bond strength (19.8 MPa). The shear bond strength between two successive SFRC layers (Group 8) was statistically (*p* < .05) the highest (43.7 MPa) of all tested groups.

**Figure 3.**
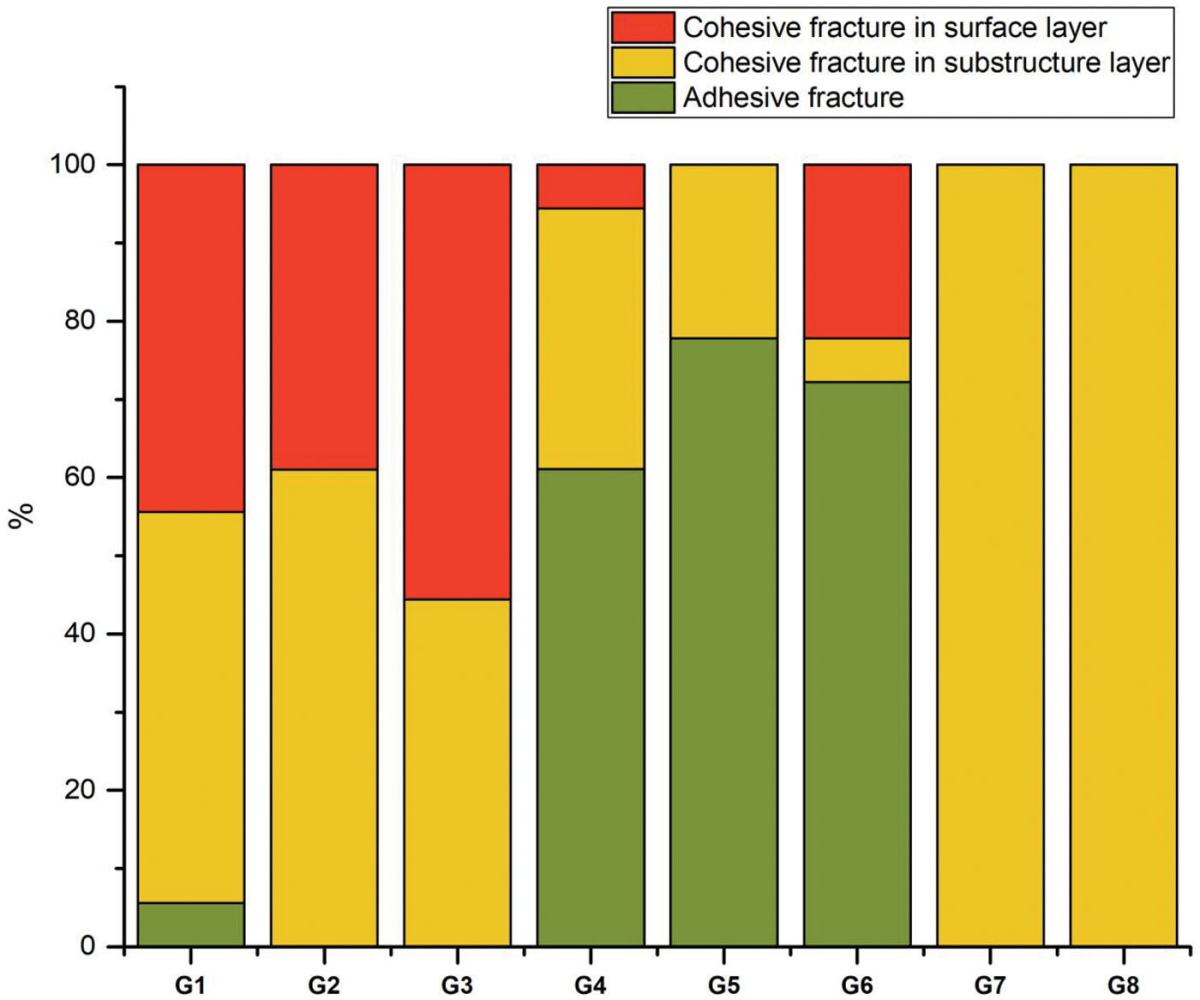
Percentage of the various failure modes in the tested groups (*n* = 18).

The failure mode results are presented in Figure 3. Ethanol-treated surfaces (Groups 2 and 3) resulted in entirely cohesive failures as did Group 1 (without surface treatment) except from one specimen, while roughening (Group 6) or treating the surface with acid etching and bonding agent (Groups 4 and 5) increased the number of adhesive failures. In groups 7 and 8, having two layers of similar material, all specimens showed cohesive failure in substructure layers.

**Figure 4.**
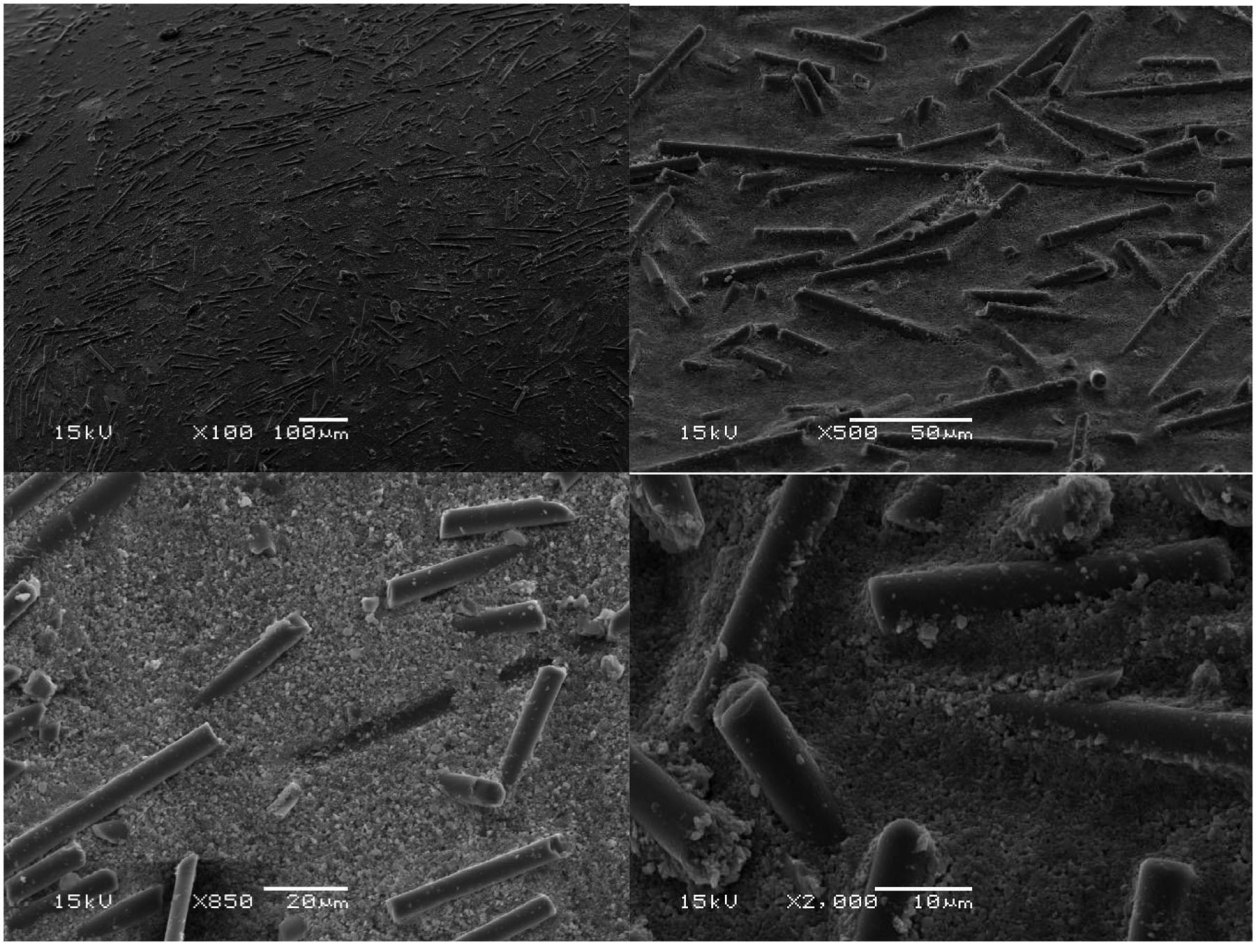
SEM images of an ethanol-treated surface of SFRC at different magnifications.

Figure 4 shows SEM images of SFRC after ethanol surface treatment under different magnifications. Ethanol treatment resulted in irregular surfaces with some short fibers protruding from the matrix.

## Discussion

Bi-layered composite restorations where flowable SFRC is placed at the cavity bottom as a substructure and veneered with PFC (packable or flowable) have been the recommended technique for restoring stress-bearing posterior teeth as they provided enhancement in load-bearing capacity when tested *in vitro* [9,16]. In this scenario, surface roughness, surface free-energy, material reactivity, viscosity, the presence of an oxygen inhibition layer, and the increment material employed all have an influence on the bonding between two composite layers [19,20].

In the current study, the existence of an oxygen inhibition layer on the surface of the cured SFRC substructure layer (without any treatment) may explain that the bond strength to the PFC surface layer (Group 1) was within the same range as that observed after using different surface treatments (Figure 2). In general, this finding is in line with many studies in the literature, in which the existence of an oxygen inhibition layer in between two successive dimethacrylate-based composites improve the interfacial bond strength [19–23]. In other words, the oxygen inhibition layer appears to act as an adhesive layer, chemically binding successive composite increments. Bijelic-Danova et al. showed that the existence of short fibers in SFRC has a beneficial effect on the thickness or depth of the oxygen inhibition layer and thus on the interfacial bonding strength [20].

**Figure 5.**
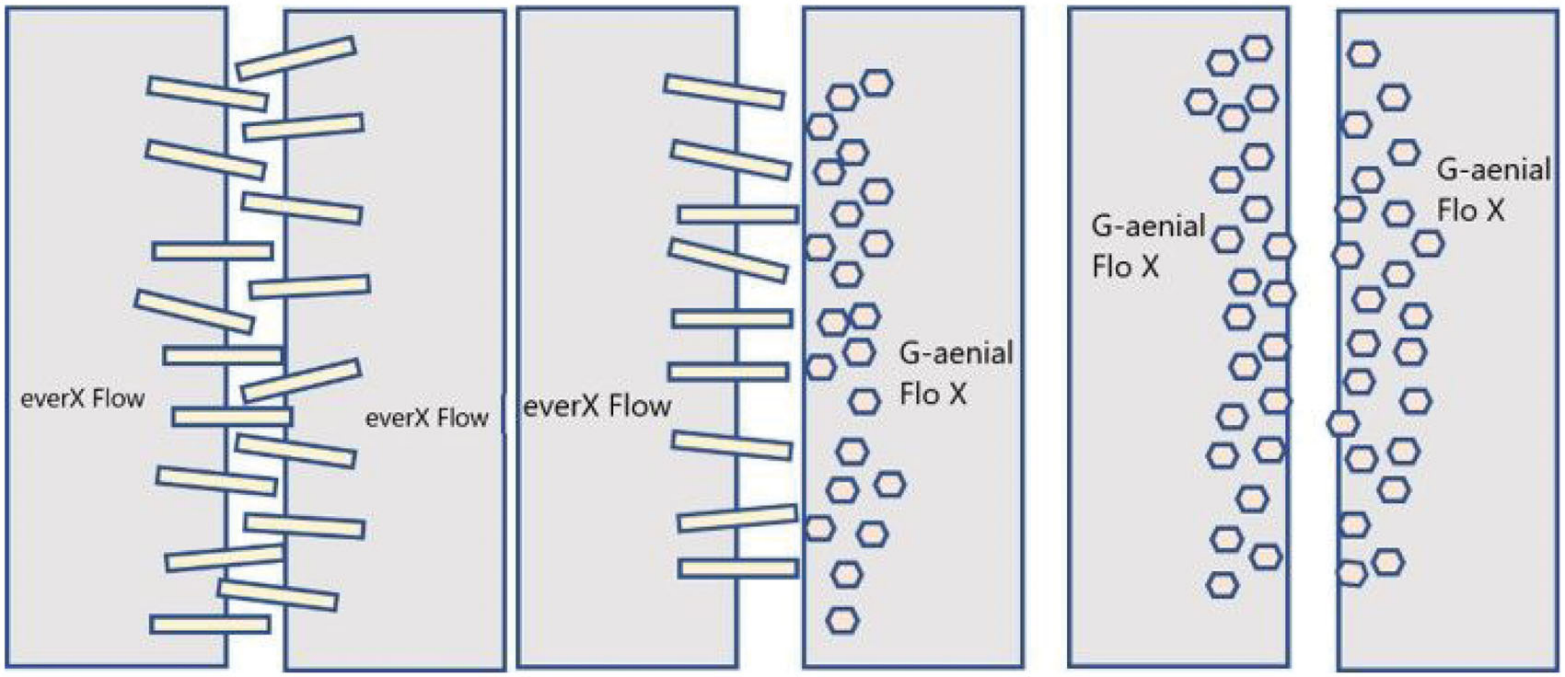
Schematic figure of the nature of interlayer surfaces between the tested materials.

Our results did not fully support the assumption that ethanol surface treatment might enhance the bond strength between SFRC and PFC layers by exposing more fibers from the surface. However, specimens in the ethanol-treated groups predominantly showed cohesive failures, which could be a sign of micro-mechanical interlocking between the monomer from PFC and the fibers in the SFRC substructure (Figure 4). In the study by Basavarajappa et al., it was found that the surface roughness of fiber-reinforced composite was influenced by ethanol at varying concentrations and treatment time [18]. This was likely related to the swelling and resolidification of the polymer surface between the glass fibers which were not affected by ethanol [18]. It is also possible that some of the residual monomers may have leached from the polymer matrix [24] and had a minor effect on the dimensions of the polymer matrix between the fibers (Figure 4). However, the orientation of the exposed fibers at the interface (Figures 4 and 5) affects the bonding and load transfer behavior. Nevertheless, this issue should be investigated further to confirm the effect in practice.

Another aspect in this study was the use of an adhesive. Groups in which an adhesive was applied between the layers (Groups 4 and 5) showed no improvement in the interfacial shear bond strength compared with Group 1 (without surface treatment), and the predominant mode of failure was adhesive (Figure 3). This result could be attributed to the brittleness caused by the existence of a relatively thick adhesive layer at the interface. Roughening the SFRC surface by grinding followed by phosphoric acid etching (Group 6), resulted in a higher shear bond strength compared to the group without surface treatment (Figure 2). This favorable finding may be explained by the resulting high surface irregularity, which increases the bonded surface area and offers higher micro-mechanical interlocking at the interface between SFRC and PFCs [25,26]. Moreover, this procedure of grinding and etching the surface with phosphoric acid could be beneficial in the case of composite repairs where there is no oxygen inhibition layer.

Our findings are in accordance with evidence from another investigation [27], which showed that treating the composite substrates with hydrofluoric acid adversely affected the morphological features of PFC substrates thereby resulting in poor repair bond strength when compared with the use of air-particle abrasion [27]. According to Özcan et al., when composite substrates are exposed to hydrofluoric acid, a water monolayer may penetrate *via* voids to the filler, which in turn, may disorganize the silane layer that is responsible for stabilizing the filler-resin interface [27]. This may weaken the particle or fiber–matrix interface that leads to filler dissolution.

There is no consensus as to a required minimum composite interlayer shear bond strength value. However, based on literature, values in the range 15 MPa to 35 MPa seem relevant [19–23,25,26,28,29]. In our study, the shear bond strength values obtained were within this range, except for the significantly highest value (43.7 MPa) found between the two SFRC layers (Group 8). This superior result could be explained by the presence of randomly orientated fibers in SFRC, which are shown to affect the oxygen inhibition depth [20,30] and by a micro-mechanical interlocking between the protruding short fibers on the interlayer surfaces (Figure 5). This interlocking could have an impact on the bond strength values, particularly in the case of shear stress. In addition, the superior mechanical properties of the SFRC, especially the fracture toughness would enhance its ability to resist shearing stresses [31,32].

The results of this investigation must be seen in the perspective of some limitations. The interlayer bond strength of composites was determined using a shear bond strength test, where the tensile-bond strength could be more accurate in detecting bond strength differences between materials [33]. However, the shear bond test set up has been the most commonly employed laboratory technique for evaluating the bond strength of adhesives and composite restorations.

Furthermore, the shear bond strength was measured without any aging, and thus long-term water storage and/or thermocycling are warranted to evaluate the long-term durability of the interlayer bonds.

Within the limitations of this study, it can be concluded that the interlayer bond strength between SFRC and PFC when an oxygen-inhibited layer is preserved, was within the same range as that observed between successive PFC layers.

## References

[1] Kopperud SE, Rukke HV, Kopperud HM, et al. Light curing procedures – performance, knowledge level and safety awareness among dentists. J Dent. 2017;58:67–73. 28179193 10.1016/j.jdent.2017.02.002

[2] Ferracane JL. Resin composite-state of the art. Dent Mater. 2011;27(1):29–38. 21093034 10.1016/j.dental.2010.10.020

[3] Manhart J, Chen H, Hamm G, et al. Buonocore memorial lecture. Review of the clinical survival of direct and indirect restorations in posterior teeth of the permanent dentition. Oper Dent. 2004;29(5):481–508. 15470871

[4] Demarco FF, Corrêa MB, Cenci MS, et al. Longevity of posterior composite restorations: not only a matter of materials. Dent Mater. 2012;28(1):87–101. 22192253 10.1016/j.dental.2011.09.003

[5] Xu HH, Quinn JB, Smith DT, et al. Effects of different whiskers on the reinforcement of dental resin composites. Dent Mater. 2003;19(5):359–367. 12742430 10.1016/s0109-5641(02)00078-7

[6] Garoushi S, Vallittu PK, Lassila LV. Short glass fiber reinforced restorative composite resin with semi-inter penetrating polymer network matrix. Dent Mater. 2007;23(11):1356–1362. 17204319 10.1016/j.dental.2006.11.017

[7] Zandinejad AA, Atai M, Pahlevan A. The effect of ceramic and porous fillers on the mechanical properties of experimental dental composites. Dent Mater. 2006;22(4):382–387. 16055180 10.1016/j.dental.2005.04.027

[8] Garoushi S, Sailynoja E, Vallittu PK, et al. Physical properties and depth of cure of a new short fiber reinforced composite. Dent Mater. 2013;29(8):835–841. 23726127 10.1016/j.dental.2013.04.016

[9] Garoushi SK, Hatem M, Lassila LVJ, et al. The effect of short fiber composite base on microleakage and load-bearing capacity of posterior restorations. Acta Biomater Odontol Scand. 2015;1(1):6–12. 28642894 10.3109/23337931.2015.1017576 PMC5433219

[10] Tiu J, Belli R, Lohbauer U. Rising R-curves in particulate/fiber-reinforced resin composite layered systems. J Mech Behav Biomed Mater. 2020;103:103537. 31765951 10.1016/j.jmbbm.2019.103537

[11] Lassila L, Keulemans F, Säilynoja E, et al. Mechanical properties and fracture behavior of flowable fiber reinforced composite restorations. Dent Mater. 2018;34(4):598–606. 29366493 10.1016/j.dental.2018.01.002

[12] Attik N, Colon P, Gauthier R, et al. Comparison of physical and biological properties of a flowable fiber reinforced and bulk filling composites. Dent Mater. 2022;38(2):e19–e30. 34961643 10.1016/j.dental.2021.12.029

[13] Lassila L, Säilynoja E, Prinssi R, et al. Characterization of a new fiber-reinforced flowable composite. Odontology. 2019;107(3):342–352. 30617664 10.1007/s10266-018-0405-y PMC6557871

[14] Molnár J, Fráter M, Sáry T, et al. Fatigue performance of endodontically treated molars restored with different dentin replacement materials. Dent Mater. 2022;38:83–93. 10.1016/j.dental.2022.02.007 35227528

[15] Lassila L, Säilynoja E, Prinssi R, et al. Bilayered composite restoration: the effect of layer thickness on fracture behavior. Biomater Investig Dent. 2020;7(1):80–85. 10.1080/26415275.2020.1770094 PMC7521310 33015638

[16] Fráter M, Sáry T, Molnár J, et al. Fatigue performance of endodontically treated premolars restored with direct and indirect cuspal coverage restorations utilizing fiber-reinforced cores. Clin Oral Invest. 2022;26(4):3501–3513. 10.1007/s00784-021-04319-3 PMC8979888 34846558

[17] Fráter M, Sáry T, Jókai B, et al. Fatigue behavior of endodontically treated premolars restored with different fiber-reinforced designs. Dent Mater. 2021;37(3):391–402. 33353735 10.1016/j.dental.2020.11.026

[18] Basavarajappa S, Perea-Lowery L, Aati S, et al. The effect of ethanol on surface of semi-interpenetrating polymer network (IPN) polymer matrix of glass-fibre reinforced composite. J Mech Behav Biomed Mater. 2019;98:1–10. 31174080 10.1016/j.jmbbm.2019.05.030

[19] Li J. Effects of surface properties on bond strength between layers of newly cured dental composites. J Oral Rehabil. 1997;24(5):358–360. 9183029 10.1046/j.1365-2842.1997.00508.x

[20] Bijelic-Donova J, Garoushi S, Lassila LV, et al. Oxygen inhibition layer of composite resins: effects of layer thickness and surface layer treatment on the interlayer bond strength. Eur J Oral Sci. 2015;123(1):53–60. 25556290 10.1111/eos.12167

[21] Truffier-Boutry D, Place E, Devaux J, et al. Interfacial layer characterization in dental composite. J Oral Rehabil. 2003;30(1):74–77. 12485387 10.1046/j.1365-2842.2003.01008.x

[22] AlJehani YA, Baskaradoss JK, Geevarghese A, et al. Shear bond strength between fiber-reinforced composite and veneering resin composites with various adhesive resin systems. J Prosthodont. 2016;25(5):392–401. 26215932 10.1111/jopr.12315

[23] Omran TA, Garoushi S, Lassila L, et al. Bonding interface affects the load-bearing capacity of bilayered composites. Dent Mater J. 2019;38(6):1002–1011. 31666485 10.4012/dmj.2018-304

[24] Basavarajappa S, Al-Kheraif AA, ElSharawy M, et al. Effect of solvent/disinfectant ethanol on the micro-surface structure and properties of multiphase denture base polymers. J Mech Behav Biomed Mater. 2016;54:1–7. 26410760 10.1016/j.jmbbm.2015.09.007

[25] Kallio TT, Tezvergil-Mutluay A, Lassila LV, et al. The effect of surface roughness on repair bond strength of light-curing composite resin to polymer composite substrate. Open Dent J. 2013;7:126–131. 24167536 10.2174/1874210601307010126 PMC3807581

[26] Mangoush E, Lassila L, Vallittu PK, et al. Shear-bond strength and optical properties of short fiber-reinforced CAD/CAM composite blocks. Eur J Oral Sci. 2021;129(5):e12815. 34322917 10.1111/eos.12815

[27] Özcan M, Alander P, Vallittu PK, et al. Effect of three surface conditioning methods to improve bond strength of particulate filler resin composites. J Mater Sci: Mater Med. 2005;16(1):21–27. 15754140 10.1007/s10856-005-6442-4

[28] Tezvergil-Mutluay A, Lassila LV, Vallittu PK. Incremental layers bonding of silorane composite: the initial bonding properties. J Dent. 2008;36(7):560–563. 18467017 10.1016/j.jdent.2008.03.008

[29] Al Musa AH, Al Nahedh HN. Incremental layer shear bond strength of low-shrinkage resin composites under different bonding conditions. Oper Dent. 2014;39(6):603–611. 24807812 10.2341/13-104-L

[30] Vallittu PK. Oxygen inhibition of autopolumerization of polymethyldimethacrylate-glass fiber composite. J Mater Sci Mater Med. 1997;8(8):489–492. 15348715 10.1023/a:1018578210453

[31] Tsujimoto A, Barkmeier WW, Takamizawa T, et al. Bonding performance and interfacial characteristics of short fiber-reinforced resin composite in comparison with other composite restoratives. Eur J Oral Sci. 2016;124(3):301–308. 26954878 10.1111/eos.12262

[32] Tsujimoto A, Barkmeier WW, Takamizawa T, et al. Relationship between mechanical properties and bond durability of short fiber-reinforced resin composite with universal adhesive. Eur J Oral Sci. 2016;124(5):480–489. 27696555 10.1111/eos.12291

[33] El Zohairy AA, Saber MH, Abdalla A, et al. Efficacy of microtensile versus microshear bond testing for evaluation of bond strength of dental adhesive systems to enamel. Dent Mater. 2010;26(9):848–854. 20569977 10.1016/j.dental.2010.04.010

